# Multiple Behavioral Risk Factors As Assets for Chronic Disease Prevention: Observations From Urban Primary Care Settings in Crete, Greece

**DOI:** 10.7759/cureus.56711

**Published:** 2024-03-22

**Authors:** Emmanouil K Symvoulakis, Panagiotis Stachteas, Emmanouil Smyrnakis, Panagiotis Volkos, Aikaterini E Mantadaki, Andreas Karelis, Chrysi Petraki, Kadiani Nioti, Stylianos Mastronikolis, Aikaterini M Antoniou, Manolis Linardakis

**Affiliations:** 1 Clinic of Social and Family Medicine, Department of Social Medicine, School of Medicine, University of Crete, Heraklion, GRC; 2 Laboratory of Primary Health Care, General Practice and Health Services Research, School of Medicine, Aristotle University of Thessaloniki, Thessaloniki, GRC; 3 Fourth Local Health Team, Academic Unit of Heraklion, Heraklion, GRC; 4 Department of Family Medicine, Private Primary Care Facilities, Heraklion, GRC; 5 Department of Ophthalmology, University of Patras, Patras, GRC; 6 Department of Respiratory Medicine, School of Medicine, University of Crete, Heraklion, GRC

**Keywords:** health profile, covid-19, perceived stress, non-communicable diseases, primary health care, behavioral risk factors

## Abstract

Aim: This study aimed to assess the health profile of patient-attendees visiting primary healthcare (PHC) practice settings in the midst of the COVID-19 pandemic and to explore the relationships between multiple behavioral risk factors (MBRFs) and consultation-driven health information. Multiple behavioral risk factors involve a variety of unhealthy behaviors that are associated with an increased prevalence of non-communicable diseases (NCDs).

Subjects and methods: The study design was based on a dataset analysis, afterward exploring the feasibility and diagnostic capacity of respiratory morbidity aspects from a study previously conducted. The study dataset contained information regarding socio-demographic characteristics, health habits, clinical information, and reported comorbidities from 183 primary care patient-attendees. A categorical regression analysis was performed, using as a numeric variable the multiple MBRFs (clustering of 0 to four factors) in order to examine relationships with the basic and clinical characteristics of the patient-attendees.

Results: Based on this secondary analysis, it was found that the prevalence of MBRFs is quite common among patient-attendees visiting urban PHC facilities. The prevalence of current smoking, sleep deprivation, increased body weight, and medium/high perceived stress levels were 33.9%, 52.5%, 83.1%, and 35.0%, respectively. An increased occurrence of MBRFs might be significantly predicted by the lower age of patient-attendees (b = -0.221, p = 0.05), by the absence of gray hair at an early age (b = -0.144, p = 0.042), by the physical discomfort during activities (b = 0.191, p = 0.017), or by the lower oxygen saturation (b = -0.184, p = 0.004). Diabetes mellitus (25.1%) was the most prevalent condition, followed by bronchial asthma (18.6%) and depression (15.8%).

Conclusions: Lower age, absence of premature hair whitening, physical discomfort during activities, and lower oxygen saturation are linked with an increased occurrence of MBRFs, leading to a neglected way of living. Those factors could be used to alert researchers, policymakers, and PHC professionals to act accordingly in order to prevent or early diagnose NCDs.

## Introduction

Lifestyle habits, or multiple behavioral risk factors (MBRFs), involve a variety of unhealthy and risky behaviors, namely, smoking, alcohol consumption, sedentary behavior, obesity, and unhealthy dietary choices. Many of these health-risk behaviors occur in combination, meaning they tend to cluster within individuals [1]. Moreover, Gidron (2013) stated that the identification of multiple risk factors allows for the treatment of individuals by assessing their significant independent risk factors [2]. They have been associated with an increased prevalence of non-communicable diseases (NCDs), like cardiovascular diseases, diabetes, cancer, and chronic respiratory diseases [3,4], which are the leading cause of morbidity and mortality worldwide [5,6]. At this point, it should be mentioned that the prevalence of MBRFs varies among populations; however, it has been observed that clustering of two or more behavioral risk factors (BRFs) (like smoking, low consumption of fruits and vegetables, rare or no physical activity, and excessive alcohol drinking) is a strong indication of poor quality of life and an increased risk for decreased life expectancy [7].

Additionally, MBRFs have been reported to be correlated with other socio-demographic and mental health factors. For instance, they have been associated with perceived stress in people living in deprived areas [8], while lower levels of subjective and objective socioeconomic status (SES) were also linked with the presence of BRFs and higher perceived stress [9]. Moreover, smoking for males and being obese for women were reported to be related to increased stress levels, while smoking was more powerfully correlated with cancer and a lack of physical activity with cardiovascular disease (CVD) mortality [10]. Based on the aforementioned findings, BRFs have complex associations that include social, psychological, demographic, and economic aspects of the population, which need to be taken into account [4, 9].

Apart from this, some of the factors linked to people’s lifestyle seem to be connected with several health conditions. In particular, smoking, sleep deprivation, stress, and premature gray hair have been associated with various health conditions, including obesity, diabetes, heart conditions, high blood pressure, stroke, and metabolic and cardiovascular risk factors [11-15]. The COVID-19 pandemic, probably due to the prolonged stay-at-home period, increased involvement with digital media, and psychological distress (anxiety, stress, and uncertainty) [16], has led to the propagation of unhealthy behaviors and the acquisition of new unfavorable lifestyle habits. Particularly during the period of strict restrictive measures (lockdown), the homebound constraint resulted in reduced physical activity, increased caloric intake, an increase in the consumption of alcohol and tobacco products, or even food precariousness [17-19]. In France, for example, a survey conducted during the first lockdown (April-May 2020) among 37.252 adults found that 53% of participants reduced physical activity, 63% increased sedentary behavior, and 35% gained weight (about 1.8 kg on average) [20]. Examining the relationship between MBRFs and NCDs from data collected in the midst of the pandemic may provide interesting information for future healthcare adaptations in addressing policy dilemmas on chronic morbidity and service sustainability.

The current study was based on a secondary analysis. Data were gathered to test case-finding feasibility for hidden respiratory morbidity between 2021 and 2022. Assembling baseline primary healthcare (PHC) information, this study aimed to assess the health profile of patient-attendees visiting urban general practice settings during the COVID-19 pandemic and to investigate the relationships between MBRFs and consultation-driven health information. Exploring MBRFs at a younger age may offer information on how to uncover health issues that have been neglected and could lead to future chronic morbidity in order to increase the competency of the PHC consultation by offering patient-centered care services.

## Materials and methods

Study design, population, and setting

Secondary data analysis is defined as “the analysis of existing data that may not have been solely gathered to focus on a particular research question” [21]. Secondary analysis provides researchers the opportunity to explore research questions utilizing available datasets, saving time and resources [21]. A primary care dataset was available from a previous study, exploring the feasibility of testing interstitial lung disease (ILD) case-finding capacity and exploring referral pathways for suspected cases [22]. Health information was collected from a cross-sectional, prospectively driven study, and participants were recruited from two private, solo primary care practices that approximately cover 5,000 inhabitants in Heraklion, Crete, Greece. As previously described [22], a person-to-person interview was carried out by healthcare professionals to obtain information regarding medical history and other health data from participants during nine months between 2021 and 2022. Before participating in the study, each patient gave written informed consent. From the 225 patients recruited, 183 formed the primary care group. One hundred and nine patients were referred.

The dataset contained information on age, gender, occupation, marital status, education, height, weight, self-reported history of gray hair at a young age (under 40 years), perceived stress levels, smoking habits, vaccination coverage, chronic disease occurrence, oxygen saturation, daily dry cough during the last six months, physical discomfort, abnormal readings at previously performed spirometry or Piko-6 readings, clinical examination components, as well as dyspnea at the current visit with the presence of crackles or finger clubbing [22]. Invited participants were primary care patient-attendees, seeking usual care services [22]. The current dataset has the entire PHC flow information before referrals, and thus the research hypothesis, sample, variable selection, and process analysis differ from those of the published, same-sourced study, which exclusively focused on referred participants [22].

Measurements and evaluation tools

Regarding the variables, age was calculated by the birth date of each patient to obtain more accurate data. Smoking habits, including the number of cigarettes smoked per day, smoking initiation, and smoking cessation age, were analyzed. Night sleep duration was self-reported. Additionally, body mass index (BMI) was estimated by height and weight using the Quetelet Index formula. Perceived stress was expressed as Perceived Stress Scale (PSS)-14 scoring. Information regarding the presence of gray hair at an early age (before the 40s) was also self-recalled. Finally, levels of physical discomfort were estimated by close-ended questions. Oxygen saturation was measured during the consultation on-site using a pulse oximeter and recorded accordingly.

Through secondary analysis of all available primary care setting data [22], four MBRFs were assessed, including smoking, increased BMI [3, 23], sleep deprivation, and stress level. Smokers were classified as those who smoke more than one cigarette per day [24]. Sleep deprivation was based on the definition of Watson et al. (2015) as having a sleep routine lasting less than seven hours per night [25]. High body weight was determined based on self-reported measurements of body weight and height as having a BMI ≥25.0 kg/m^2^ (i.e., being overweight or obese) [3,4,24]. The stress level was defined using the PSS-14, a tool with reliable psychometric quality validated for use in the Greek adult population [26-28]. The PSS-14 is not a screening tool for stress and therefore does not involve cut-offs; however, a score limit of 25-26 was considered a moderate or high level of stress for the current sample. Moreover, gender, age, gray hair at an early age, physical discomfort during activities, and oxygen saturation were included in the categorical regression analysis.

To estimate the clustering of MBRFs, each factor was coded separately into a binary variable (0: absence, 1: presence). The prevalence of clustering was then estimated by adding the binary variables, generating a clustering score ranging from 0 to four. ‘Multiple clustering of 2+ factors’ was considered to depict a higher risk for chronic diseases [3,4,24].

Statistical analysis

The data analysis was effectuated using IBM SPSS Statistics for Windows, version 25.0 (IBM Corp., Armonk, NY). The frequency distributions of the explicit characteristics of the participants were calculated. The prevalence of morbidity and the four MBRFs were also evaluated based on 95% confidence intervals (95% CIs). Additionally, the observed (O) and expected (E) prevalence of individual and MBRF clusters were assessed to estimate O/E ratios and provide the most frequent combinations (clusters) of the four MBRFs. Finally, categorical regression analysis was performed, using as a numeric variable the multiple MBRFs (clustering of ‘0’, ‘1’, ‘2’, '3', or ‘4’ factors) to examine relationships with the basic and clinical characteristics of patient-attendees within a PHC setting. All relevant parameters were used as ordinal predictors in optimal scaling, estimating standardized beta coefficients (b), their standard errors (SE), and the relative importance of predictive parameters (Pratt index). Multicollinearity was also evaluated by the high negative values of significance as well as by the tolerance values of independent parameters that exceeded 0.80.

Ethical considerations

Ethics approval was granted by the Research Committee of the University of Crete (approval number: 30.11.2020/205), in accordance with the Helsinki Declaration.

## Results

**Figure 1:**
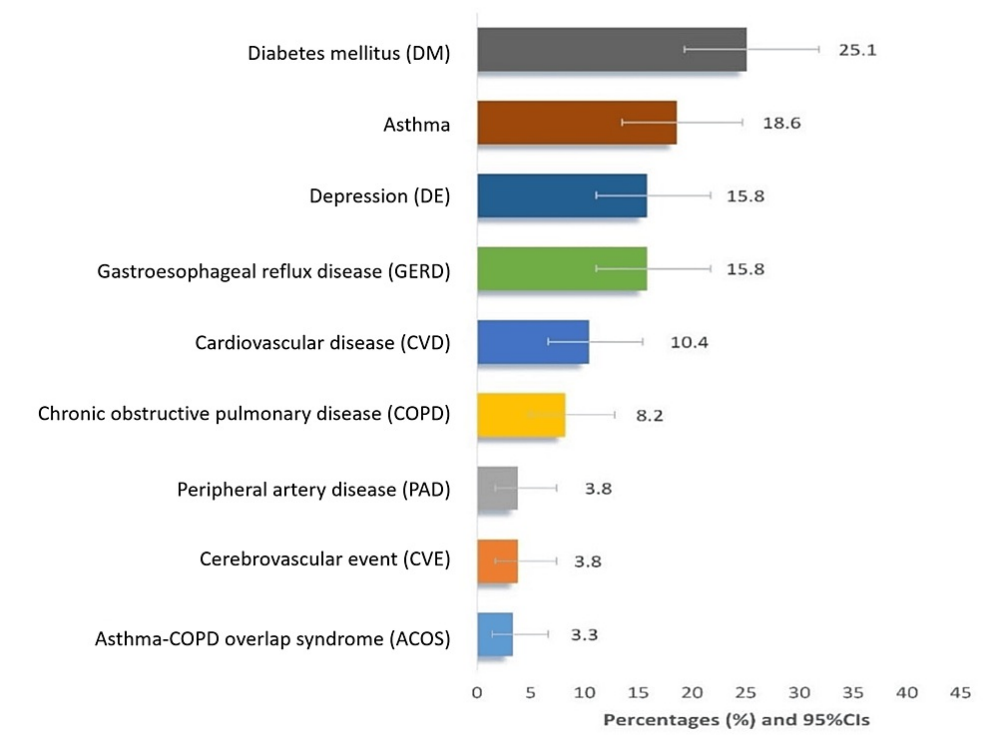
Current disease frequency of 183 patient-attendees in primary health care settings included in the study

Data from 183 primary care participants were used for the current analysis. The mean age of participants was 62.2 (SD 9.0) years, and notably, 91.3% (n = 167) of them were above 50 years of age, and most were women (54.6%, n = 100). Table 1 presents the baseline characteristics of the 183 patient-attendees within the PHC setting of the current study. Regarding the various chronic conditions, 44.8% (n = 82) of the participants reported having no NCDs, whereas the prevalence of comorbidity (3+ conditions) was found in 10.9% (n = 20) of the patients. Nevertheless, diabetes mellitus (25.1%, n = 47) was the most prevalent condition, followed by bronchial asthma (18.6%, n = 35) (Figure 1).

**Table 1:** The baseline characteristics of 183 patient-attendees from the primary care setting of the current study stand. dev.: standard deviation

Particulars		n	%
Gender	Male	83	45.4
	Female	100	54.6
Age (years)	Mean ±stand. dev. (min, max)	62.2±9.0 (37.3, 88.9)	
	50+	167	91.3

Regarding the MBRFs of the participants, ex- or current smokers represented 44.3% of participants (n = 81) and 33.9% (n = 62), respectively (Table 2). The mean consumption of cigarettes was estimated to be 40.8 pack-years. The mean sleeping duration was found to be 6.5 (SD = 1.4) hours, the mean BMI was 29.6 (SD = 5.4) kg/m^2^, and remarkably, only 16.5% (n = 30) of patient-attendees had a normal BMI. The mean PSS was 19.3 (SD = 6.9) and the mean oxygen saturation was 96.7% (SD = 1.7). Having gray hair at an early age (before the 40s) was reported by 17.5% (n = 32) of participants. Physical discomfort during activities was reported by 34.6% (n = 63) of the participants, and discomfort at rest was reported by 12.6% (n = 23); discomfort in both situations was reported by 12.1% (n = 22).

**Table 2:** Health habits and physical and mental health levels of 183 patient-attendees from the primary care setting of the current study stand. dev.: standard deviation; BMI: body mass index; PSS-14: perceived stress scale; COPD: chronic obstructive pulmonary disease; ACOs: asthma-COPD overlap syndrome; GERD: gastroesophageal reflux disease; CVD: cardiovascular disease; DE: depression; DM: diabetes mellitus; CVE: cerebrovascular event; PAD: peripheral artery disease

Particulars			n (%)
Smoking		Non-smoker	40 (21.9)
		Ex-smoker	81 (44.3)
		Currently smoking	62 (33.9)
Packyears		Mean (median) (min, max)	40.8 (30.0) [0.1, 265.0]
Night-time sleep hours		Mean±stand. dev. (min, max)	6.5±1.4 (2.0, 10.0)
BMI (kg/m^2^)		Mean±stand. dev. (min, max)	29.6±5.4 (14.4, 50.6)
		Underweight (BMI <18.5)	1 (0.5)
		Normal (BMI 18.5-24.9)	30 (16.5)
		Overweight (BMI 25.0-29.9)	76 (41.5)
		Obese (BMI 30.0+)	76 (41.5)
Perceived Stress Scale (PSS)-14		Mean±stand. dev. (min, max)	19.3±6.9 (0, 40)
	Score 22-56 or medium/high stress level		64 (35.0)
Oxygen saturation (in rest) (%)		Mean±stand. dev. (min, max)	96.7±1.7 (91, 99)
		≤95	32 (17.9)
Grey hair at an early age (before 40s)		No	151 (82.5)
		Yes	32 (17.5)
Physical discomfort during activities		During rest	23 (12.6)
		During daily physical activity	63 (34.6)
		None	118 (64.8)
		During one	42 (23.1)
		During both	22 (12.1)
Comorbidity (chronic conditions such as COPD, Asthma, ACOS, GERD, CVD, DE, DM, CVE, PAD)		None	82 (44.8)
		1	60 (32.8)
		2	21 (11.5)
		3+	20 (10.9)

Among the 183 patient-attendees, the prevalence of current smoking, sleep deprivation, high body weight, and medium/high perceived stress level was 33.9% (n = 62), 52.5% (n = 96), 83.1% (n = 152) and 35.0% (n = 64), respectively (Table 3). Interestingly, only 2.7% (95% CI: 1.1-4.9) of patient-attendees had no risk factors, while 4.9% (95% CI: 2.5-8.8) had all four risk factors. Notably, 71.1% (95% CI: 64.0-77.2) of participants had 2+ risk factors. In the clustering of MBRFs, the smoking habit individually seemed to be more prevalent than expected or had the greatest degree of clustering, as the observed prevalence was found to be 147% higher than the expected one (O÷E = 2.47) and was followed by the combination of smoking and sleep deprivation (Ο÷Ε=2.24).

**Table 3:** The prevalence of four multiple behavioral risk factors (MBRFs) among 183 patient-attendees within the primary care setting of the current study ^a^ 95% CIs: 95% confidence intervals ^b^ body mass index (BMI) ≥25.0 kg/m^2^

Particulars		n	%	95% CIs^a^
Smoking, current smokers	No	121	66.1	59.1, 72.7
	Yes	62	33.9	27.3, 40.9
Sleep deprivation, <7 hours/night	No	87	47.5	40.4, 54.8
	Yes	96	52.5	45.2, 59.6
High body weight, overweight & obese^b^	No	31	16.9	12.0, 22.9
	Yes	152	83.1	77.1, 88.0
Medium/high stress level, the score of Perceived Stress Scale 22+	No	119	65.0	57.9, 71.7
	Yes	64	35.0	28.3, 42.1
Clustering of multiple behavioral risk factors (MBRFs)	0 (none)	5	2.7	1.1, 5.9
	1	48	26.2	20.3, 32.9
	2	73	40.0	33.0, 47.1
	3	48	26.2	20.3, 32.9
	4	9	4.9	2.5, 8.8
	2+	130	71.1	64.0, 77.2

Regression analysis of the relationship between the clustering of MBRFs and the demographic and clinical characteristics of patient-attendees (Table 4), showed that the higher presence of MBRFs could be significantly predicted by the lower age of patient-attendees (b = -0.221, p = 0.05), by the absence of gray hair at an early age (b = -0.144, p = 0.042), by the physical discomfort during activities (b = 0.191, p = 0.017), or by the lower oxygen saturation (b = -0.184, p = 0.004). According to the relative significance of predictors, oxygen saturation and physical discomfort during activities had the highest cumulative contribution (62.2%) to the presence of MBRFs, while age and gray hair at an early age contributed by 32.2%.

**Table 4:** Categorical regression analysis to investigate the relationship between clustering of multiple behavioral risk factors (MBRFs) and the basic and clinical characteristics of 178 patient-attendees within the primary care setting of the current study ^a^ The numeric variable of multiple BRFs (clustering of ‘0’, ‘1’, ‘2’, ‘3’ or ‘4’ factors) was used as a categorical dependent variable.

Particulars	Clustering of multiple behavioral risk factors (MBRFs) ^a^				
Parameters	Standardized beta (b)	Standard error	F	p-value	Importance (Pratt index)
Gender (1: males, 2: females)	-0.028	0.073	0.15	0.698	-0.004
Age (years)	-0.221	0.077	8.22	0.005	0.190
Comorbidity (1: no chronic condition, 2: one, etc.)	0.079	0.072	1.22	0.272	0.062
Gray hair at an early age (before the 40s) (1: no, 2: yes)	-0.144	0.070	4.21	0.042	0.132
Physical discomfort during activities (1: none, 2: in one, 3: in both)	0.191	0.079	5.81	0.017	0.300
Oxygen saturation (%)	-0.184	0.063	8.53	0.004	0.322
R^2^ (adjusted)	0.122 (0.091)				

## Discussion

Based on this secondary analysis, it was found that the prevalence of MBRFs is quite common among patients visiting urban PHC facilities. Notably, seven out of 10 participants had more than two risk factors, which suggests that the health profile of patients visiting primary care facilities, which occurred in the midst of the pandemic, was curiously interesting. Regression analysis of the relationship between clustering of MBRFs and basic and clinical characteristics of patient-attendees revealed that the presence of MBRFs could be significantly predicted by the lower age of patient-attendees, not having gray hair at an early age, experiencing more physical discomfort during activities, or having lower oxygen saturation.

The prevalence of unhealthy behaviors was apparent in this sample, which is in agreement with earlier studies in similar population groups. In a study among European adults aged 50 years or older, the presence of MBRFs was quite common, and the most prevalent risk factor was overweight or obesity in men and physical inactivity in women, while the prevalence of two or more risk factors was higher in men [4]. Previous data in the literature suggested that men, generally, had a higher prevalence of MBRFs for NCDs [29-32]. Notwithstanding, no significant differences in the prevalence of risk factors were observed between genders in the current results. The 2001 National Health Interview Survey, concerning adults aged 18 years or older living in the United States, also found that people with two, three, or four risk factors had significantly higher odds of having a chronic disease [30]. In the present study, the most prevalent disease was diabetes mellitus; however, regression analysis of the relationship between the clustering of MBRFs and the basic and clinical characteristics of patient-attendees showed that the presence of MBRFs was not predicted by comorbidity (the presence of one or more NCDs).

On the other hand, it has been revealed that sufficient levels of physical activity have generally been shown to be protective against the development of several NCDs, including cancer [33], but may also contribute positively to mental health status by alleviating levels of anxiety or depression [11]. In this study, high BMI was the most prevalent risk factor among the participants, a fact that is in accordance with other studies showing that being overweight or obese is very common among adults. The aforementioned population group had a higher incidence of chronic diseases like cardiovascular disease or cancer and a higher mean number of symptoms of aging, such as pain in the knees (and associated risk factors such as back or hip pain) [34].

Moreover, a larger local general practice study showed that 651 out of 802 primary care users were overweight or obese [35], while data from 11 European countries earlier supported that 60% of the participants were also overweight or obese [4]. Interestingly, it was also reported that in 70 out of 195 participating countries, the prevalence of obesity doubled between 1980 and 2015, while it increased in the majority of the rest of the countries [36].

Furthermore, the fact that the current study was conducted during the COVID-19 isolation measures in Greece needs to be considered when discussing the findings since this time may have influenced the health profile of patient-attendees. The COVID-19 outbreak occurred worldwide and triggered unprecedented changes in all aspects of life. Restrictive measures such as social distancing, quarantine, lockdowns, and mask mandates were adopted by many countries around the world to mitigate virus spread and had a negative effect on the daily habits of people [37]. As a result, many alterations were noted in terms of people’s behaviors, such as a decrease in physical activity levels, a significant increase in sedentary behavior and caloric intake, an increase in the consumption of alcohol and tobacco products, a tendency toward non-quality food consumption [17], and an increase in body weight [38]. In Greece, with the exception of leisure physical activity, which significantly increased, all other forms of physical activity decreased significantly, with an overall decline of 16.3% [19]. Based on the above, it could be supposed that some behavioral risk factors that were also assessed in the present survey were negatively affected by the restrictive measures. Attention is needed on how to approach behavioral, but potentially modifiable, risk factors, in order to adjust excess hazards during crises. Future research could focus on exploring how behavioral risk factors are evolving in the years after the COVID-19 pandemic.

These findings are crucial for emphasizing the need for national agencies, stakeholders, policymakers, health systems, and professionals to identify ways to address and prevent the consequences of MBRFs. According to some studies, high taxation on tobacco products, alcohol, and foods with high energy density [39], clean indoor air laws, and advertisement constraints or mass media campaigns promoting healthy lifestyle behaviors are some other interventions that could contribute to mitigating the prevalence of some risk factors [40,41].

In the PHC setting, health promotion initiatives should identify ways and interventions to reduce MBRF prevalence, focus on patients with multiple risk factors, and offer them individualized care and guidance according to personal motivations, skills, and impediments to lifestyle changes [42,43]. Such interventions may be applied by the interdisciplinary PHC team either in the community or at primary healthcare facilities. Timely identification of MBRFs may not be a simple task, as is expected, in everyday-burden PHC settings. Our findings suggest that factors such as lower age, not having gray hair at an early age, having more physical discomfort during activities, or having lower oxygen saturation may be seen as an easy and non-time-consuming tool to predict MBRFs’ likelihood and act to ‘intercept’ a silent event cascade towards NCDs early. However, further studies, focusing on a longitudinal design and incorporating a larger number of participants, are needed to elucidate the predictive value of these factors in PHC.

Strengths and limitations

The present study describes an interesting attempt to produce primary research findings by analyzing available data through purposeful methodological tips. First of all, using existing databases, the interface between morbidity and health can be further explored. Additionally, by investigating associations between behavioral or not conventionally used lifestyle determinants, an opportunity to explain aspects of current or prospective morbidity in a primary care environment appears to be a viable pathway. This complementary input possibly aligns research and clinical observations in the context of 'real locality’ studies.

Our study also has various methodological limitations. The present analysis was based on a cross-sectional design and therefore cannot be sustained as a causal relationship. In the current study, the impact of the pandemic is certainly indirect, but it cannot be ignored. In addition, due to the heterogeneity of the literature regarding MBRFs, it was not possible to compare pre-and post-pandemic findings with the present study’s results.

Also, it was conducted solely in two primary care centers in Crete and included a small sample of patients. Consequently, our results are not likely to mirror the health needs of the patients seeking primary care at urban general practice centers in Greece; additional studies in community health centers and in rural locations are needed. Moreover, the assessment of smoking prevalence was merely based on current smoking occurrence at the time of study and not on the total burden of this behavior (e.g., pack-years). The sample composition, in terms of gender, was found to be in agreement with a previous observation that women are slightly more frequent users of PHC services at a local level [35].

Furthermore, self-reported information may always be influenced by participants since it cannot be confirmed, especially for issues of the past. Therefore, variables such as gray hair at an early age (before the 40s) and an assessment of physical discomfort might be misreported. Additionally, other factors such as body weight or height were also self-reported, so they may be overestimated or underestimated by participants and differ from objective measurements. Ideally, future studies should include a validation of self-reported data by conducting objective measurements in a subsample of the population, when possible.

## Conclusions

This is an exploratory, prospective study designed on mixed lifestyle and primary care-sourced information to assess the health profile of patient-attendees visiting private urban general practice settings during the COVID-19 pandemic and to overall interlink primary care reporting with behavioral habits and bio-info features of attendees. Lower age and the absence of premature hair whitening, both linked to an increased occurrence of MBRFs, may yield a perception of “invulnerability”, leading to a neglected health lifestyle. The majority of the participants reported having at least two of the behavioral risk factors that could be used to alert researchers, policymakers, and PHC professionals to develop appropriate interventions. Lastly, other factors such as lower age, not having gray hair at an early age, having more physical discomfort during activities, or having lower oxygen saturation may be utilized as an easy and non-time-consuming tool to identify people with MBRFs and act accordingly to prevent or early diagnose NCDs.
